# Multiplex Digital Quantification of β-Lactamase Genes in Antibiotic-Resistant Bacteria by Counting Gold Nanoparticle Labels on Silicon Microchips

**DOI:** 10.3390/bios12040226

**Published:** 2022-04-09

**Authors:** Galina V. Presnova, Denis E. Presnov, Anna A. Filippova, Ilia I. Tsiniaikin, Mariya M. Ulyashova, Maya Yu. Rubtsova

**Affiliations:** 1 Department of Chemistry, Lomonosov Moscow State University, 119991 Moscow, Russia; gkovba@enzyme.chem.msu.ru (G.V.P.); iiffii@mail.ru (A.A.F.); umarusya@mail.ru (M.M.U.); 2 D.V. Skobeltsyn Institute of Nuclear Physics, M.V. Lomonosov Moscow State University, 119991 Moscow, Russia; denis.presnov@physics.msu.ru; 3 MSU Quantum Technology Centre, 119991 Moscow, Russia; ii.tcinyaykin@physics.msu.ru; 4 Cryoelectronics Lab, Faculty of Physics, M.V. Lomonosov Moscow State University, 119991 Moscow, Russia

**Keywords:** high-sensitive digital detection, quantification, functionalized gold nanoparticles, microchips, β-lactamases, antibiotic-resistant bacteria

## Abstract

Digital quantification based on counting of individual molecules is a promising approach for different biomedical applications due to its enhanced sensitivity. Here, we present a method for the digital detection of nucleic acids (DNA and RNA) on silicon microchips based on the counting of gold nanoparticles (GNPs) in DNA duplexes by scanning electron microscopy (SEM). Biotin-labeled DNA is hybridized with capture oligonucleotide probes immobilized on the microchips. Then biotin is revealed by a streptavidin–GNP conjugate followed by the detection of GNPs. Sharp images of each nanoparticle allow the visualization of hybridization results on a single-molecule level. The technique was shown to provide highly sensitive quantification of both short oligonucleotide and long double-strand DNA sequences up to 800 bp. The lowest limit of detection of 0.04 pM was determined for short 19-mer oligonucleotide. The method’s applicability was demonstrated for the multiplex quantification of several β-lactamase genes responsible for the development of bacterial resistance against β-lactam antibiotics. Determination of nucleic acids is effective for both specific DNA in lysates and mRNA in transcripts. The method is also characterized by high selectivity for single-nucleotide polymorphism discrimination. The proposed principle of digital quantification is a perspective for studying the mechanisms of bacterial antibiotic resistance and bacterial response to drugs.

## 1. Introduction

Modern progress in nucleic acids (DNA and RNA) detection techniques has a great impact on biomedical applications in diagnostics of bacterial and viral infections, genetic predispositions to disorders, food safety, anti-bioterrorism, and others [1,2,3]. Among these tasks, the diagnosis of infections caused by various microorganisms is one of the primary areas in clinical diagnostics. According to the WHO, bacterial infections represent one of the leading causes of death in developing countries [4]. Despite the presence of a variety of antibacterial drugs, the widespread presence of strains resistant to their action limits options for effective therapy [5,6]. Major challenges concern the early diagnosis of the etiology of infections and their susceptibility to antibiotics. Over the last decade, many biosensor platforms have been developed to detect different bacterial and viral markers [7]. Among them, fast and label-free technologies based on opto-photonic, electro-photonic, and Raman spectroscopy have been developed for real-time monitoring and primary typing of bacteria at the level of single cells [8,9,10]. Novel methods have been proposed for studying biofilms as communities of surface-attached bacterial cells which are more tolerant to antibiotics compared to planktonic cells [11]. Various methods involving arrays of micro- and nanoelectrodes combined with optoelectronic devices; arrays of interdigitated electrodes combined with impedance spectroscopy; and the method of resonant hyperspectral imaging have been reported for monitoring the initial bacterial attachment to control biofilm formation [12,13,14].

A more detailed characterization of bacteria and their resistance to antibiotics is carried out using molecular typing of nucleic acids. Recent developments involve methods providing multiplexing capability and miniaturization (DNA-based nanobiosensors, biobarcode assays and DNA microchips) [15,16,17]. The main focus of genetic methods is achieving high sensitivity. For this purpose, single-molecule techniques for detecting molecular interactions are being actively developed [18], and great attention is drawn to digital detection based on single-molecule counting, which is considered one of the most powerful tools for ultrasensitive biomarker identification [19].

Digital counting of single molecules can be carried out using various high-resolution imaging techniques. Among them, scanning electron microscopy (SEM) is being effectively applied for the visualization and characterization of various biological objects from 1 nm in size, involving cells, viruses, bacteriophages, etc. [20,21]. SEM is also actively used to evaluate biosensor surface morphology and characteristics [22,23,24]. However, its use for the direct analysis of nucleic acids is difficult due to their small size. To overcome this limitation, indirect detection of nanoparticles as molecular tags for biomolecular complexes can be used. Metal nanoparticles, including gold nanoparticles (GNPs), have attracted considerable interest in various molecular nanotechnological applications involving the detection of pathogens [25,26,27]. Single-particle counting of fluorescent magnetic nanospheres was used for ultrasensitive multiplexed identification of influenza viruses with a detection limit of 0.02 pg/mL [28]. The application of a field-emission SEM was demonstrated for the quantification of single-strand model DNA of 46 bases in the format of a two-step sandwich hybridization assay on plastic microchips by visualization and counting of GNPs [29].

This study aimed to develop multiplex digital quantification of nucleic acids (oligonucleotides, DNA and RNA) by allele-specific hybridization on silicon microchips via the visualization and counting of GNP labels with SEM. To reduce the effect of the label on the formation of DNA duplexes, DNA targets were labeled with biotin, which was then detected in DNA duplexes by a conjugate of streptavidin with GNPs.

β-Lactamase genes were selected as model DNAs, the multiplex determination of which is extremely relevant for clinical diagnosis. β-Lactamases are responsible for the development of bacterial resistance to β-lactam antibiotics (penicillins, cephalosporins, carbapenems, monobactams), which are the most widely used in clinical practice [30,31]. These enzymes are extremely diverse, including serine and metallo-enzymes, and are divided into four molecular classes (A, B, C, and D) according to their structure [32]. Multi- and pan-antibiotic-resistant bacteria carry many resistance genetic determinants, including several types of β-lactamase genes [33], which explain the need to develop multiplex methods to determine them. For this work, we have selected four relevant β-lactamases genes: TEM-1, CTX-M-3, CTX-M-5 (molecular class A), and VIM-1 (molecular class B). Identification was performed in DNA lysates isolated from bacterial cultures as well as in RNA transcripts to explore the possibility of applying this technique to determine β-lactamase gene expression. We also studied the applicability of the developed method for determining single-nucleotide polymorphism (SNP) in genes. In class A β-lactamases, certain SNPs encode substitutions of key amino acids that lead to changes in substrate specificity for cephalosporins. Two of them were selected for identification by digital counting of GNP labels.

We optimized the conditions for DNA hybridization analysis using a highly specific biotin–streptavidin reaction for labeling DNA duplexes with GNPs and the conditions for their visualization and counting by SEM. To determine long DNA molecules, the conditions for biomolecules’ unfolding were optimized to increase the efficiency of hybridization. The analytical parameters for the quantification of short oligonucleotides and long DNAs corresponding to the genes of four types of β-lactamases were determined. Total DNA and RNA isolated from bacterial cells were used as samples of nucleic acids. The high specificity of SNP determination in β-lactamase genes was also shown.

## 2. Materials and Methods

### 2.1. Materials

All chemicals and organic solvents were of analytical grade. dNTPs, *Taq* DNA polymerase, and dUTP-11-Biotin were obtained from Fermentas (St. Leon-Rot, Germany); 3-glycidopropyl trimethoxysilane (GPTMS), chloroauric acid, mercaptosuccinic acid, dithiothreitol, ethylenediaminetetraacetic acid (EDTA), sodium sulfate, sodium chloride, sodium dodecyl sulfate (SDS), bovine serum albumin (BSA), casein, Tween-20, toluene, methanol, and isopropanol were purchased from Sigma-Aldrich (St. Louis, MO, USA). Sodium citrate dihydrate was purchased from MP Biomedicals (Eschwege, Germany). Streptavidin was obtained from Imtek (Moscow, Russia). All water used in experiments was purified with a Milli-Q system (Millipore, Billerica, MA, USA).

Primers and capture oligonucleotide probes containing a 5′-amino group and 13-mer thymidine spacer were synthesized and purified by Syntol (Moscow, Russia). Their sequences are listed in Table 1.

GNPs with a diameter of 25 nm were prepared by the Frens method [34], based on the reduction of chloroauric acid by sodium citrate. A conjugate of streptavidin with GNPs was prepared by covalent binding of streptavidin modified with mercaptosuccinic acid with the colloidal solution of GNPs as described earlier [35].

### 2.2. Silicon Microchips Fabrication

The surface of the silicon plates was purified with oxygen plasma for 30 min using a reactive-ion etching technique (Alcatel RDE-300, 30 Pa, 25 Wt) and was then treated with 10 mM GPTMS in dry toluene for 12 h at 80 °C, followed by washing for 10 min at 100 °C [36]. Further, samples of modified silicon were stored in highly pure alcohol.

Capture oligonucleotide probes (20 μM) dissolved in 0.25 M Na-phosphate buffer, containing 0.3 M Na_2_SO_4_, were spotted onto the silicon microchips with XactII Microarrayer (Lab Next Inc., Glenview, IL, USA). The microchip fabrication scheme and the layout of the capture probes are shown in Figure S1 (Supplementary Materials). Each probe was spotted in triplicate. After the immobilization, the microchips were blocked using a solution of 1% BSA and 1% casein in 10 mM K-phosphate buffer, pH 7.2, containing 0.15 M NaCl.

### 2.3. Amplification and Labeling of Target DNA

DNA samples isolated from four phenotypically and genotypically characterized control and laboratory strains (*E. coli* EPI300, producing β-lactamase CTX-M-3; *E. coli* TOP10/STY20, producing β-lactamase CTX-M-5; *E. coli* J53, producing β-lactamase TEM-1; *Ps. aeruginosa* VR-143/97, producing β-lactamase VIM-2) were kindly provided by Dr. M.V. Edelstein (Institute of Antimicrobial Chemotherapy of Smolensk State Medical Academy, Smolensk, Russia).

Total bacterial DNA was extracted with the InstaGene Matrix Kit (Bio-Rad, Hercules, CA, USA) and used as a template for PCR amplification. Total RNA was extracted with the «RNA-Extran» Kit (Syntol, Moscow, Russia) by the method of acidic phenol extraction. cDNA synthesis was carried out via the reverse transcription reaction using «OT-1» Kit (Syntol, Moscow, Russia). Samples of cDNA were then used as a template for PCR amplification.

Target DNA was amplified by the PCR and labeled with biotin by introducing dUTP-11-biotin during the reaction. The PCR was carried out in a total volume of 25 μL containing 1.0 μL of bacterial DNA in PCR buffer (2.5 mM MgCl_2_, 50 mM KCl, 10 mM Tris-HCl, pH 8.3), 0.5 μM of direct and reverse primers, 100 μM (each) dATP, dGTP, and dCTP; 60 μM dTTP; 40 μM dUTP-11-biotin and 2.5 U of Taq DNA polymerase [37]. The amplification was performed in a Mastercycler gradient thermal cycler (Eppendorf AG, Hamburg, Germany) accordingly to the protocol: an initial denaturation step (94 °C for 2 min) was followed by 20 cycles, each consisting of 94 °C for 20 s, 60 °C for 30 s, and 72 °C for 45 s, and a final extension step at 72 °C for 4 min. The quantity and molecular weight of PCR products were estimated by horizontal electrophoresis in 1% agarose gel.

### 2.4. Hybridization of Target DNA on Silicon Microchips

The microchips were placed in the wells of a 48-well polystyrene plate (Greiner, Germany) and hybridized with biotinylated target oligonucleotide (Probe C) or DNA resuspended in 2xSSPE buffer (0.04 M NaH_2_PO_4_, 0.6 M NaCl, 4 mM EDTA, 6 mM MgCl_2_, pH 7.4). Hybridization was carried out in a Thermomixer Comfort (Eppendorf AG, Hamburg, Germany) for 2 h at 45 °C. After the hybridization, the microchips were washed once with 2xSSPE containing 0.2% SDS at 45 °C for 10 min and then twice with PBST (10 mM K-phosphate buffer, pH 7.2, containing 0.15 M NaCl and 0.1% Tween-20) for 10 min at room temperature. To identify SNPs, long target DNA was fragmented prior to the hybridization by DNase I (0.5 mU per 1 ng of DNA) to yield 50 to 150 bp fragment sizes.

### 2.5. Digital Detection and Data Processing

The microchips were incubated in a solution of the streptavidin–GNPs conjugate in PBST containing 1% BSA at 37 °C for 30 min, washed twice with PBST for 10 min, once with H_2_O for 3 min, and dried on air. The GNPs on the silicon surface were detected using a Supra-40 field emission scanning electron microscope (Carl Zeiss AG, Jena, Germany) with an InLens secondary electron detector built in the microscope column. The accelerating voltage and beam current were adopted to achieve the best resolution and contrast for GNP visualization. The number of GNPs on microchip fragments was counted using the Gwyddion software (Czech Metrology Institute, Brno, Czech Republic). The limit of DNA detection (LOD) was calculated as the mean number of GNPs registered for a blank DNA sample (0 pM DNA) plus 2 standard deviations (SD, *n* = 10).

## 3. Results and Discussion

### 3.1. Assay Principle, Counting of Gold Nanoparticles on Silicon Microchips

**Figure 1:**
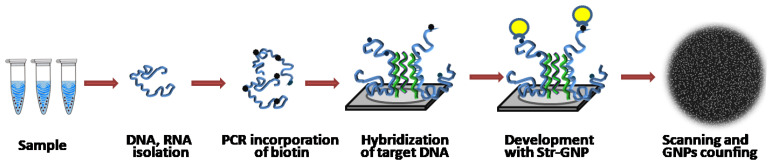
Scheme of successive stages of DNA hybridization analysis on microchips with digital detection of gold nanoparticles.

The experimental procedure is shown schematically in Figure 1. Standard silicon wafers with orientation <100> were chosen for the fabrication of microchips. The choice of the material was determined by its compatibility with all microelectronic processes and availability. A semiconductor silicon crystal is characterized by low conductivity, which is sufficient to avoid charge effects during the process of silicon sample imaging in an electron microscope. As a result, images with a resolution up to 1 nm are obtained. Since, under normal conditions, the surface of silicon is covered with a layer of native silicon oxide ~2–3 nm thick, it was treated with oxygen plasma to increase the number of free hydroxyl groups for subsequent chemical modification using GPTMS.

**Figure 2:**
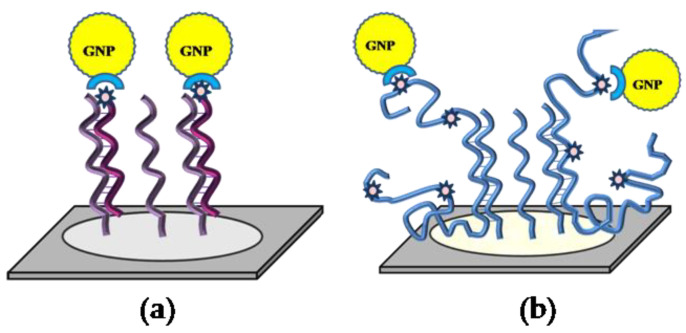
Schematic representation of hybridization of biotin-labeled short target oligonucleotides (**a**) and long target DNA (**b**) on silicon DNA microchips. 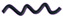
—capture oligonucleotide probes immobilized on the microchip surface, 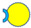
—a conjugate of streptavidin with gold nanoparticles, 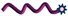
—target oligonucleotides labeled with biotin, 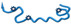
—target DNA labeled with biotin.

Capture oligonucleotide probes with 13 T spacer and amino-group at 5′-end were covalently immobilized on modified silicon microchips (Figure S1). Biotin-labeled short oligonucleotides or target DNA were hybridized on the microchips, and then biotin labels in DNA duplexes were developed by a conjugate of streptavidin with GNPs (Figure 2). The conjugate was obtained by covalent attachment of streptavidin modified with mercaptosuccinic acid to GNPs, since this method was shown to improve the signal-to-noise ratio substantially [35]. After washing and drying, SEM images of the microchips were registered and analyzed.

**Figure 3:**
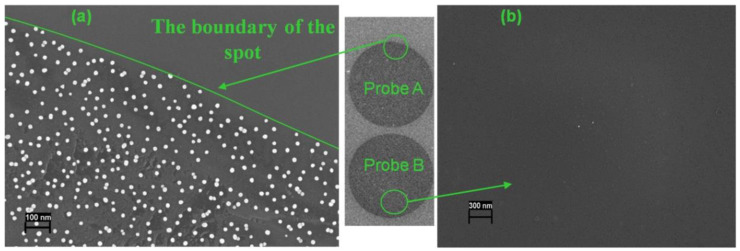
SEM images of the specific microchip spots after hybridization of 300 pM target Probe C with complementary Probe A (**a**) and non-complementary Probe B (**b**). Biotin in DNA duplexes was developed with a streptavidin–GNPs conjugate.

First, we studied the hybridization of the model biotin-labeled 19-mer oligonucleotide (Probe C) with complementary (Probe A) and non-complementary (Probe B) capture oligonucleotide probes immobilized on the microchip. SEM images of specific (Probe A) and control (Probe B) microchip spots after interaction with streptavidin-GNPs conjugate are shown in Figure 3. Nanoparticles were detected clearly by SEM on the spots with complementary Probe A, and their distribution was uniform. Each recorded nanoparticle is referred to as one duplex molecule, as each target oligonucleotide contains one biotin label. So, a sharp image of each nanoparticle allows the visualization of hybridization results on a single-molecule level. A significant difference was observed between the number of GNPs registered on the specific and control microchip spots. Only trace amounts of GNPs were registered on the control spot and the areas outside of the spots with the complementary Probe A as well.

Then, the SEM was applied for the characterization of long DNA hybridization. Two target DNAs were studied: 507 bp DNA, coding a large fragment of *bla*_VIM-1_, and 870 bp DNA, coding the whole-length *bla*_CTX-M-3_. Several capture oligonucleotide probes were spotted on the microchip surface: two specific probes (Probe VIM and Probe CTX-M) and Probe B as a negative control. After hybridization with the target DNA, biotin labels were revealed by the conjugate streptavidin–GNPs as described above, followed by the registration of GNPs with SEM. Similarly to the hybridization of the short oligonucleotide, individual nanoparticles were recorded clearly on SEM images of the spots with complementary duplexes of target DNA and specific immobilized probes. The number of nanoparticles registered on the negative control spots was negligible.

Long DNA biopolymer molecules were present in the hybridization medium in a folded conformation, depending on the ionic strength and salt concentration. Since it may have reduced the hybridization effectiveness, we investigated the addition of magnesium chloride and SDS to promote their unfolding. The effect of MgCl_2_ and SDS concentrations on the GNP density registered on the specific spots is shown in Figure S2 (Supplementary Materials). The number of particles detected was dependent on the concentration of magnesium ions, and the dependence was bell-shaped (Figure S2a). Although the flatness of the curve maximum depended on the type of gene, we chose a concentration of 6mM magnesium ions, at which the number of nanoparticles on the surface increased as much as possible. The effect of SDS was also characterized by the dependence of a similar form (Figure S2b). The increased number of formed DNA duplexes was observed at a wide range of SDS concentrations, and it did not depend on the DNA type. However, SDS concentrations above 7.5 mM led to an uneven distribution of the detected DNA duplexes; accordingly, the standard deviation of the number of registered particles increased also.

**Figure 4:**
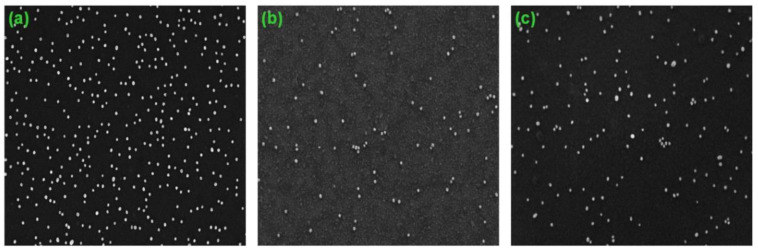
SEM images of the specific spots after hybridization of the microchip with 0.8 nM (**a**) and 60 pM (**b**) target DNA, containing blaCTX-M-3, and 60 pM target oligonucleotide Probe C (**c**).

Labeling of long DNA molecules in our method is based on the incorporation of biotin–dUTP during the PCR, and each DNA amplicon contains several biotin labels (data not shown). Their further development with the conjugate of streptavidin with GNPs may result in several nanoparticle labels per DNA strand. Figure 4 shows SEM images obtained after hybridization of the microchips with different concentrations of target DNA, containing *bla*_CTX-M-3_. The image for high DNA concentrations contains closely spaced nanoparticles and their agglomerates (Figure 4a). In contrast the image for low DNA concentration (Figure 4b) is comparable with that for short oligonucleotides (Probe C), containing one biotin molecule (Figure 4c). In this case, we did not observe a large number of neighboring nanoparticles or their conglomerates for comparable concentrations of short and long DNA molecules, and the distribution of GNPs was similar. Most likely, the large size of the nanoparticles represents a steric hindrance to the interaction of several conjugate molecules with one molecule of the target DNA. Thus, it can be assumed that the visualization of GNPs as labels by SEM provides single-molecule analysis for both short and long target DNA molecules.

### 3.2. Analysis of Cleaved Silicon Microchips

**Figure 5:**
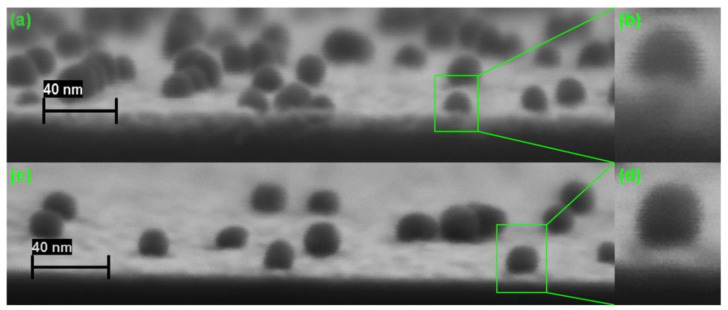
Cross-sectional SEM images of the microchip spots obtained at 80° angle after hybridization of target oligonucleotide or DNA and development of the duplexes with a streptavidin–GNP conjugate: (**a**) 80 pM target Probe C, (**b**) an enlarged image of one GNP of the duplex with short oligonucleotide, (**c**) 50 pM target DNA containing blaCTX-M-3, (**d**) an enlarged image of one GNP of the duplex with blaCTX-M-3.

In order to analyze the thickness and structure of biospecific layers formed on the silicon surface after the hybridization and development steps, the microchips spots were split up along the silicon crystallographic axis near the center of the spot, and the sample edge was explored by placing it closer to the objective lens of the microscope. This enabled us to obtain images of the surface at different angles, including the orthogonal edge view. Figure 5 shows the cross-sectional SEM images of the microchips obtained at 80° angles after hybridization of the short oligonucleotide (Probe C) and 870 bp DNA (*bla*_CTX-M-3_). It is clearly seen that the surface location of GNPs is different depending on the DNA length. In the case of short oligonucleotide duplexes, nanoparticles are recessed in the surface layer of the support formed by DNA duplexes, immobilized probes, proteins, and GPTMS (an enlarged image of one nanoparticle is shown in Figure 5b). However, in the case of large DNA duplexes, nanoparticles are located on the silicon surface (an enlarged view of one nanoparticle is shown in Figure 5d).

Differences in the surface localization of nanoparticles when analyzing short and long DNA targets can be explained as follows. The length of the capture probes, including the 13-mer spacer, is about 9–10 nm. The layer of blocking proteins on the microchip surface is non-uniform in height and is about 10–15 nm (this was shown by us earlier in another study). Thus, oligonucleotide duplexes with short capture probes (about 6 nm in size) are located very close to or even inside the protein layer, and the nanoparticles in them appear to be partially immersed in this layer. When the capture probes interact with DNA targets of 500 bp or more, the length of which is more than 170 nm, the size of the duplexes exceeds the protein layer, and GNPs appear on its surface.

SEM detects secondary electrons, passing through a thin non-conductive layer; the method is effective in the detection of all GNPs irrespective of their location on the surface of a microchip. In this regard, SEM has certain advantages over atomic force microscopy (AFM), which detects only objects placed above the surface and cannot detect reliably the nanoparticles immersed in the surface layer of the support in whole or in part [38].

### 3.3. Optimization of Digital Analysis of DNA Duplexes by SEM

Counting GNP labels was used for quantitative analysis of the short target 19-mer oligonucleotide, assuming that one nanoparticle corresponds to one DNA duplex. In order to optimize the technical parameters of SEM for better performance, counting nanoparticles at various microscope magnifications was investigated (Figure S3 in Supplementary Materials). Each magnification corresponds to a fixed frame size: 2.8 μm^2^, 11.3 μm^2^, and 52 μm^2^ for the magnifications of 150 KX, 75 KX, and 35 KX, respectively.

To study the reproducibility, the GNPs were counted on several frames on each spot. Table 2 contains the number of GNPs and variation coefficients (CV) estimated for different quantities of frames, labeled probe concentration, and microscope magnification. The most accurate values of the number of GNPs with low CVs were obtained at frames of larger size; at the same time, the resolution of individual nanoparticles was worse. At low Probe C concentrations, the particles were distributed less uniformly, which led to an increase in the CVs of up to 29% at high magnification. The optimum microscope magnification for the GNP counting was revealed to be 75 KX with CVs not more than 5–8% depending on the probe concentration. An average of three frames is sufficient to count the number of nanoparticles with good reproducibility.

For the characterization of GNP distribution in different spots, it is convenient to estimate the value of the particle density (D), which is defined as the number of nanoparticles registered per area unit:

D = Xav/S(1)

where X_av_—the average number of GNPs registered in the frame and S—the area of the frame.

D values, depending on microscope magnification and probe concentration, are shown in Table 2. This parameter does not depend strongly on microscope magnification and can be used for quantitative analysis of hybridization results.

Optimization of the hybridization protocol and washing procedure has allowed us to significantly reduce non-specific conjugate binding resulting in low values of GNPs recorded on the control spot (noise value). This value did not exceed 1–2 GNPs per frame at a magnification of 75KH.

### 3.4. Digital Quantification of Nucleic Acids on Silicon Microchips

**Figure 6:**
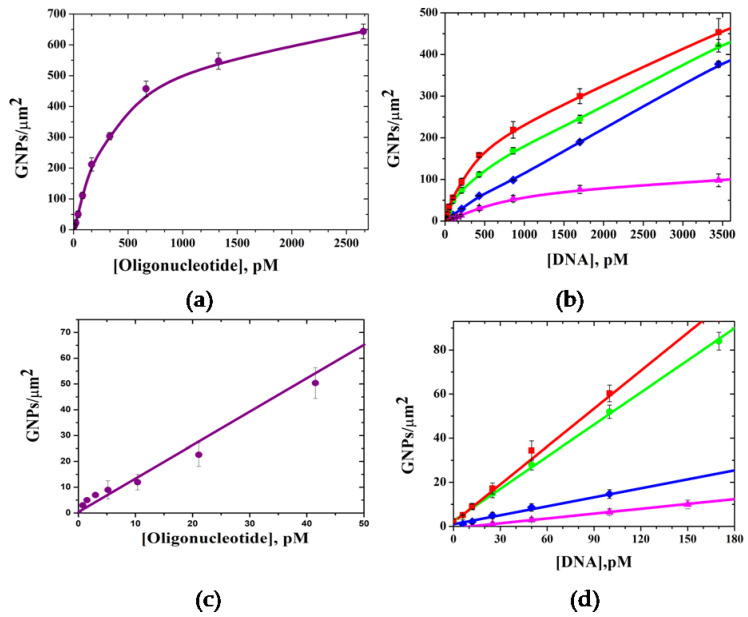
Calibration curves for the determination of short oligonucleotide Probe C (**a**) and long DNAs (**b**) containing blaTEM-1(▲), blaCTX-M-3(●), blaVIM-2 (♦), blaCTX-M-5 (■), on silicon microchips with digital detection. (**c**,**d**)—enlarged sections of the calibration curves (**a**,**b**) for low concentrations of target DNAs.

To study the feasibility of GNP digital counting by SEM for the quantitative detection of target DNA, various concentrations of short single-strand oligonucleotide (Probe C) and fragmented long double-strand DNA, containing genes of β-lactamases (*bla*_TEM-1_, *bla*_CTX-M-3_, *bla*_CTX-M-5_, *bla*_VIM-2_), were hybridized on the microchips. It was found that the density of GNPs increased with the target DNA concentration (Figure 6). The lower LOD of 0.04 pM was determined for the short 19-mer oligonucleotide (Figure 6a,c). It is one order of magnitude higher compared to previously published data for digital detection of the GNPs in sandwich hybridization of short oligonucleotides with the capture probes, which in turn was shown to be three orders of magnitude more sensitive than fluorescence detection [13]. This demonstrates the advantages of incorporating nanoparticles into DNA duplexes using a highly specific biotin–streptavidin interaction, which reduces steric hindrances. The use of streptavidin conjugated with nanoparticles makes it possible to use one universal approach for multi analysis, which was demonstrated by the determination of several β-lactamase genes on a single microchip (Figure 6b,d).

Among long double-strand DNA targets, the lower LOD of 0.3 pM was determined for *bla*_CTX-M-5_ and LODs of 0.5 pM, 5.0 pM, and 16 pM were determined for *bla*_CTX-M-5_, *bla*_VIM-2_, and *bla*_TEM-1_, respectively. The variation in the LODs for different genes is mainly due to differences in the properties of the capture probes, leading to various hybridization stringencies with the target DNAs.

Table 3 presents the data on the quantitative determination of DNA by various methods proposed recently. Most of them use GNPs as a label for DNA duplexes, which are incorporated either directly into oligonucleotide probes or using the streptavidin–biotin interaction. Several methods are based on the application of other labels (magnetic nanoparticles and enzymes). Among them, there are methods for determining both types of DNA: short (no more than 50 bases) and long (more than 150 bases). Our method with GNP digital detection is more sensitive in determining short oligonucleotide targets when compared to standard target concentration determination. The indirect method of incorporating GNPs into duplexes via the streptavidin-biotin interaction reduces the LOD by one order of magnitude compared to the digital detection of nanoparticles incorporated directly into detecting oligonucleotide probes.

All methods for determining longer DNA fragments show worse sensitivity. Presumably, this is due to the steric problems of DNA target interaction with capture and detection probes. Our method is characterized by sensitivity, comparable to the best analytical performance of other methods, while it should be noted that the longest DNA fragment was determined in our study. When compared to hybridization analysis of the same genes based on the same capture probes on DNA microchips with colorimetric detection, the LOD of our method is more than three orders of magnitude lower; also, the dynamic range is 1–2 orders of magnitude wider [42]. Comparing the analytical characteristics of the determination of short and long DNA fragments, it can be assumed that the lengthening of the capture probes may contribute to an increase in the sensitivity of the method.

The size of the detected genes is important for increasing the specificity of the analysis and reducing false positives in the diagnosis of infectious diseases. The method of real-time PCR widely used for these purposes is based on the amplification of relatively short fragments of genes. Even though our method is longer in time and more complex, one of the advantages concerns the amplification of long DNA, which was shown by the amplification of full-size β-lactamase genes.

**Figure 7:**
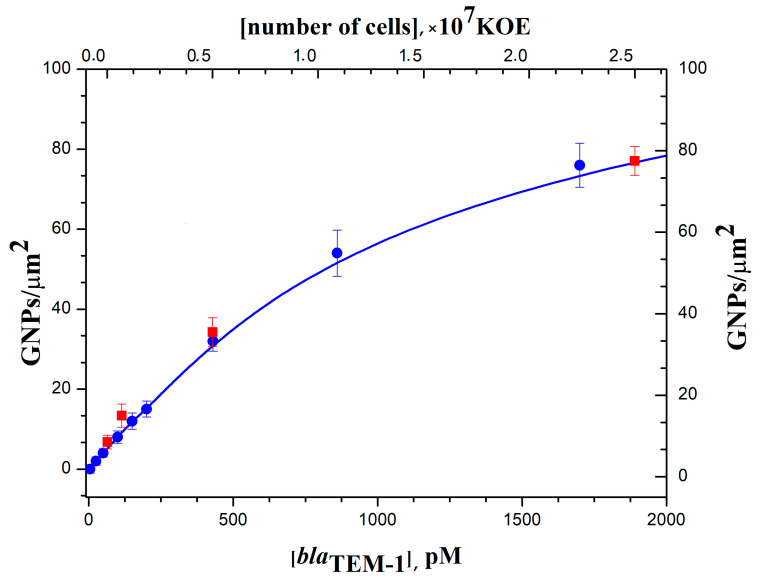
Determination of specific mRNA of the blaTEM-1 in RNA transcripts isolated from *E. coli* cells by sequential reverse transcription and PCR followed by hybridization and digital detection of GNPs (red). Calibration curve for the determination of blaTEM-1 (blue).

The method of digital nucleic acid quantification by SEM was also applied for the detection of expressed β-lactamase genes in RNA transcripts isolated from cultivated bacteria. Sample preparation for the hybridization stage involved total RNA isolation from *E. coli* cells — producers of recombinant β-lactamase TEM-1, reverse transcription of specific mRNA β-lactamase to cDNA followed by PCR similar to the preparation of target DNA from DNA lysates. Several samples of total RNA isolated from bacterial cultures containing different cell concentrations (from 2.5 × 10^4^ to 6.2 × 10^7^ CFU/mL) were prepared. From them, samples of labeled target DNA, containing *bla*_TEM-1_, were obtained in sequential reverse transcription and PCR reactions. Calibration curves for the quantitative determination of the *bla_TEM-1_* in RNA transcripts isolated from *E. coli* cells by sequential reverse transcription and PCR followed by hybridization and digital detection of GNP tags are shown in Figure 7. All calculated values of GNP density corresponded well with the calibration curve for the quantification of the *bla*_TEM-1_ gene amplified from DNA lysates. Thus, the developed hybridization analysis of specific mRNA on the DNA microchips with digital detection can be used for the identification of expressed β-lactamase genes in antibiotic-resistant bacteria. Determining the expression levels of beta-lactamase genes is important when studying the mechanisms of multi-resistance and the response of bacteria to the action of drugs.

### 3.5. Digital Identification of Single Nucleotide Polymorphism

**Figure 8:**
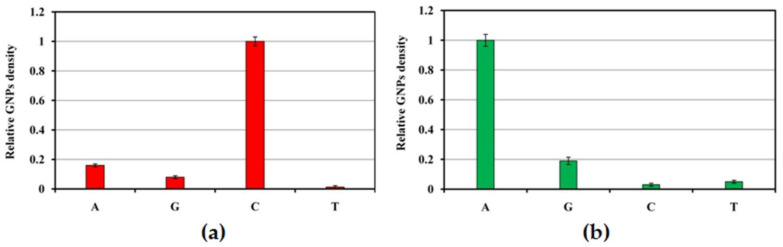
Relative GNP density determined after the hybridization of 20 ng blaCTX-M-3 target DNA with the probe sets for identification of SNPs encoding amino acid replacement at position 167 (**a**) and 240 (**b**) in β-lactamases of the CTX-M-1 subcluster. A, G, C, or T indicates a central nucleotide in the probe.

Determination of nucleic acids for diagnostic purposes often includes the identification of SNPs responsible for mutations in encoded proteins and enzymes [46]. This task is extremely important in the characterization of bacteria resistant to β-lactam antibiotics since the key substitutions in bacterial β-lactamases result in the enhanced substrate specificity of the enzymes (extended-spectrum β-lactamases, ESBLs) [31,32]. We demonstrated the capabilities of the microchips with SEM-based digital detection in identifying the SNPs encoding two key mutations of amino acid residues (P167 and D240) in β-lactamases belonging to the CTX-M-1 subcluster. Identification is based on an allele-specific hybridization approach, which exploits the differences in thermal stability of a fully complementary DNA duplex and duplexes with a single mismatch. For the detection of the SNP, a set of four probes with identical sequences except for the nucleotide at the central (or close to central) base, which was A, T, G, or C, was used (Table 1). After hybridization with target DNA and digital detection, the density of GNPs was determined for each probe of the SNP set. The probe showing the highest signal value in the result of complementary hybridization was considered the perfect match (PM); the remaining ones with lower signal values were regarded as mismatches (MM). To investigate the discriminatory power of the method, the relative GNP densities were defined by normalizing to the net GNP density of the PM probe. Figure 8 represents relative GNP densities determined after the hybridization of 20 ng *bla*_CTX-M-3_ target DNA with the probe sets for identification of SNPs encoding amino acid replacement at positions 167 (a) and 240 (b) in β-lactamases of the CTX-M-1 subcluster. Nucleotide triplets in the antisense strain of *bla*_CTX-M-3_ contain dGTP and dTTP in the positions, corresponding to amino acids P167 and D240. They were identified successfully on the microchips by complementary hybridization with the capture probes CTX-M-3_167_C and CTX-M-3_240_A, which correspond to the sense strand and include dCTP and dATP in the central position (Table 1). In both cases, identification of nucleotide type was characterized by high discriminatory power, and mismatch signals, which show the level of nonspecific hybridization, were no more than 15% of the PM signals.

## 4. Conclusions

In this study, we developed a new technique for nucleic acids quantification based on digital counting of GNP labels by high-resolution SEM. It combines the advantages of nanoparticles as labels for biospecific interactions and the ability of SEM to produce a sharp image of the microchip surface with distinguishable GNPs. GNP counting is used for the digital detection of single DNA duplexes formed on the surface of a microchip. Immobilization of capture probes of different specificity on a microchip enables multiplex determination. Indirect labeling of DNA duplexes via biotin-streptavidin interaction minimizes steric hindrances and makes it possible to analyze both short oligonucleotides and long DNA molecules of several hundred bases in length. The method is characterized by enhanced sensitivity and high signal-to-noise values.

The method of nucleic acid analysis on silicon microchips is characterized by high stability. It is ensured by the high stability of microchips with covalently immobilized oligonucleotide probes (they retain their properties for more than one year when stored at RT) and high stability of DNA duplexes and duplexes after the introduction of GNPs and washing, since streptavidin conjugate with nanoparticles was obtained by covalent cross-linking.

The method showed high sensitivity in determining both short oligonucleotides and long DNA molecules up to 800 bp. We have shown its application in the identification of full-size genes of β-lactamases, bacterial enzymes responsible for the development of resistance to β-lactam antibiotics. Such an approach also provides highly selective identification of SNPs, which is important for the identification of extended-spectrum β-lactamases. When combined with a reaction of reverse transcription, the method allows the determination of specific mRNAs of β-lactamases in RNA transcripts.

We believe that our technique may be applicable for fundamental studies of antibiotic resistance mechanisms, the development of multi-resistance in bacteria, their response to the action of drugs, and monitoring of β-lactamase genes.

## Figures and Tables

**Table 1:** Oligonucleotide probe sequences used in this work.

Probe Name	Sequence, 5′-3′	Length, Bases
Probe A	GATTGGACGAGTCAGGAGC	19
Probe B	TTCTAGACAGCCACTCATA	19
Probe C	Biotin-GCTCCTGACTCGTCCAATC	19
Probe CTX-M	ATATCGCGGTGATCTGGCC	19
Probe TEM	CCAGAAACGCTGGTGAAAGT	20
Probe VIM	GTGGTTGTGCCGTTCAT	17
CTX-M-3_167_A	GACCGTACCGAG**A**CGACGTTAAAC	24
CTX-M-3_167_G	GACCGTACCGAG**G**CGACGTTAAAC	24
CTX-M-3_167_C	GACCGTACCGAG**C**CGACGTTAAAC	24
CTX-M-3_167_T	GACCGTACCGAG**T**CGACGTTAAAC	24
CTX-M-3_240_A	GGCAGCGGTG**A**CTATGGCAC	20
CTX-M-3_240_G	GGCAGCGGTG**G**CTATGGCAC	20
CTX-M-3_240_C	GGCAGCGGTG**C**CTATGGCAC	20
CTX-M-3_240_T	GGCAGCGGTG**T**CTATGGCAC	20

**Table 2:** Counting average GNP number (X_av_), coefficients of variation (CV) and particle density (D) on separate frames of specific spots of the microchips by SEM at various microscope magnifications.

	MF *	150 KX			75 KX			35 KX		
NF **										
		X_av_	CV%	D	X_av_	CV%	D	X_av_	CV%	D
Concentration of Probe C, 5 pM										
3		24	29	8	77	8.9	7	302	4.6	6
6		23	27	8	76	5,9	7	296	3.1	6
9		23	22	8	77	4.9	6	302	2.4	6
Concentration of Probe C, 500 pM										
3		1193	3.5	426	4599	3.7	407	20, 956	3.1	403
6		1190	2.7	425	4567	2.9	412	21,060	2.3	405

*—Microscope magnification; **—Number of frames for GNPs counting.

**Table 3:** Analytical performances of different methods developed for quantitative determination of DNA.

Method/Detection Principle	DNA Target Size	Label	Limit of Detection	Reference
Determination of short oligonucleotides				
Hybridization on silicon microchips/counting of GNP labels	Oligonucleotide (19 b)	Indirect labeling of DNA duplexes with GNPs via streptavidin-biotin interaction	0.04 pM	This work
Sandwich hybridization on plastic microchips/counting of GNP labels	Oligonucleotide (46 b)	Direct labeling of detection oligonucleotide probe with GNPs	1 pM	[18]
Sandwich hybridization on the microelectrodes/detection of conductivity	Oligonucleotide (27 b)	Direct labeling of detection oligonucleotide probe with GNPs, silver enhancement	0.5 pM (500 fM)	[39]
DNA hybridization with PNA probes/colorimetric detection of GNPs	Oligonucleotide (18 b)	Electrostatic interaction of DNA duplexes with GNPs, gold or silver enhancement	10 pM	[40]
Hybridization on DNA microarrays/scanometric detection with optical scanner)	Oligonucleotide (21 b)	Labeling of the ds-DNA with DNA intercalator (daunorubicin) conjugated to GNPs, enhancement of the GNPs	10 pM	[41]
Determination of long DNA				
Hybridization on silicon microchips/counting of GNP labels	Full-size gene of β-lactamase *bla*_CTX-M-5_ (870 bp)	Indirect labeling of DNA duplexes with GNPs via streptavidin-biotin interaction	0.3 pM	This work
Hybridization on membrane microchips/colorimetric detection	Full-size gene of β-lactamase *bla*_CTX-M-5_ (870 bp)	Indirect labeling of DNA duplexes with horseradish peroxidase via streptavidin-biotin interaction	0.71 nM (0.40 ng μL^−1^)	[42]
Hybridization on biosensor array/detection of magnetoresistive ratio	Synthetic ssDNA (167 p) GAPDH gene (the fragment size is not specified)	Indirect labeling of DNA duplexes with magnetic NPs via streptavidin-biotin interaction	39 pM 0.1–1 pM depending on amount of amplification cycles	[43,44]
Sandwich hybridization on glass microchips/optical detection	Fragment of Hepatitis E virus RNA (DNA target of 500 bp)	Direct labeling of detection oligonucleotide probe with nano-gold, silver enhancement	0.1 pM (100 fM)	[45]

## Data Availability

Research data are not shared.

## Supplementary Materials

The following supporting information can be downloaded at: https://www.mdpi.com/article/10.3390/bios12040226/s1, Figure S1: The microchip fabrication scheme and the layout of the capture probes; Figure S2: Effect of Mg^2+^ and SDS concentrations on the GNPs’ density on specific microchip spots corresponding to immobilized Probe A and Probe CTX-M after the hybridization of *bla*_CTX-M-3_ and *bla*_CTX-M-5_; Figure S3: SEM images of the specific microchip spots after hybridization with 500 pM short target Probe C obtained at different magnifications of the microscope.

## Abbreviations

AFM: atomic force microscopy; BSA, bovine serum albumin; GNP, gold nanoparticle; GPTMS, 3-glycidopropyl trimethoxysilane; EDTA, ethylenediaminetetraacetic acid; SEM, scanning electron microscopy; SNP, single nucleotide polymorphism; SDS, sodium dodecyl sulfate.
