# Supplementary material for: Multiplex Digital Quantification of β-Lactamase Genes in Antibiotic-Resistant Bacteria by Counting Gold Nanoparticle Labels on Silicon Microchips
Source: Biosensors (Basel). 2022 Apr 9;12(4):226. doi: 10.3390/bios12040226 (PMC9024738; doi:10.3390/bios12040226)
Supplement: Supplementary file 1 [file biosensors-12-00226-s001.zip › biosensors-1667743-supplementary.pdf]

Supplementary material

Multiplex Digital Quantification of β-Lactamase Genes in Antibiotic-Resistant Bacteria by Counting Gold Nanoparticle Labels on Silicon Microchips

Galina V. Presnova [1], Denis E. Presnov [2,3,4], Anna A. Filippova [1], Ilia I. Tsiniaikin [3,4], Mariya M. Ulyashova [1] and Maya Yu. Rubtsova [1,*]

[1] Department of Chemistry, Lomonosov Moscow State University, 119991 Moscow, Russia; gkovba@enzyme.chem.msu.ru (G.V.P.); iiffii@mail.ru (A.A.F.); umarusya@mail.ru (M.M.U.)
[2] D.V. Skobeltsyn Institute of Nuclear Physics, M.V. Lomonosov Moscow State University, 119991 Moscow, Russia; denis.presnov@physics.msu.ru
[3] MSU Quantum Technology Centre, 119991 Moscow, Russia; ii.tcinyaykin@physics.msu.ru
[4] Cryoelectronics Lab, Faculty of Physics, M.V. Lomonosov Moscow State University, 119991 Moscow, Russia
* Correspondence: myr@enz.chem.msu.ru

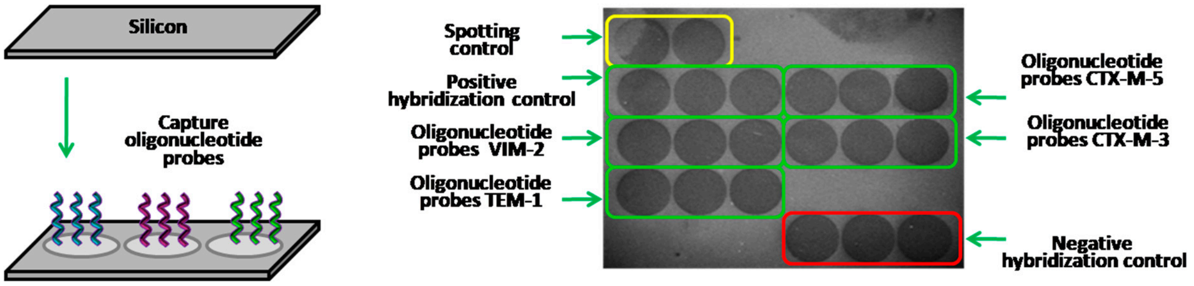

Figure S1. The microchip fabrication scheme and the layout of the capture probes.

(a)

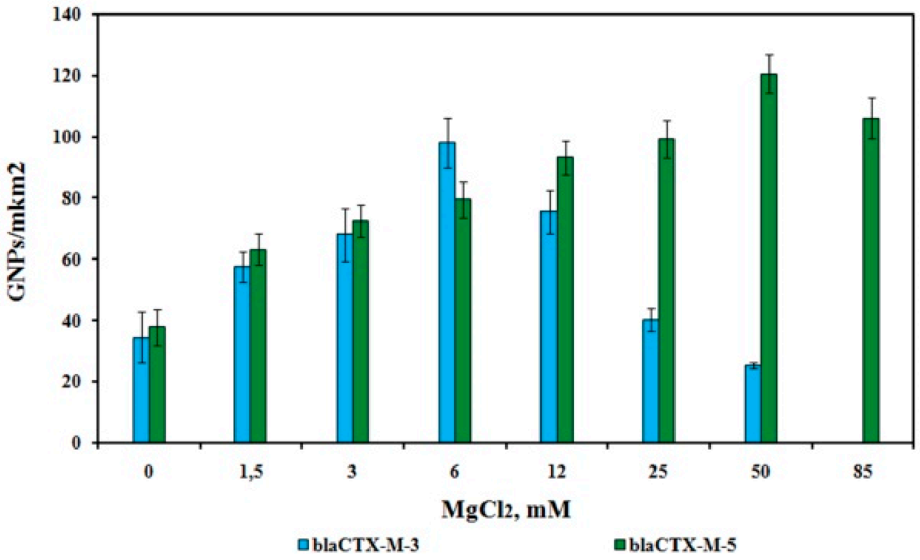

(**b**)

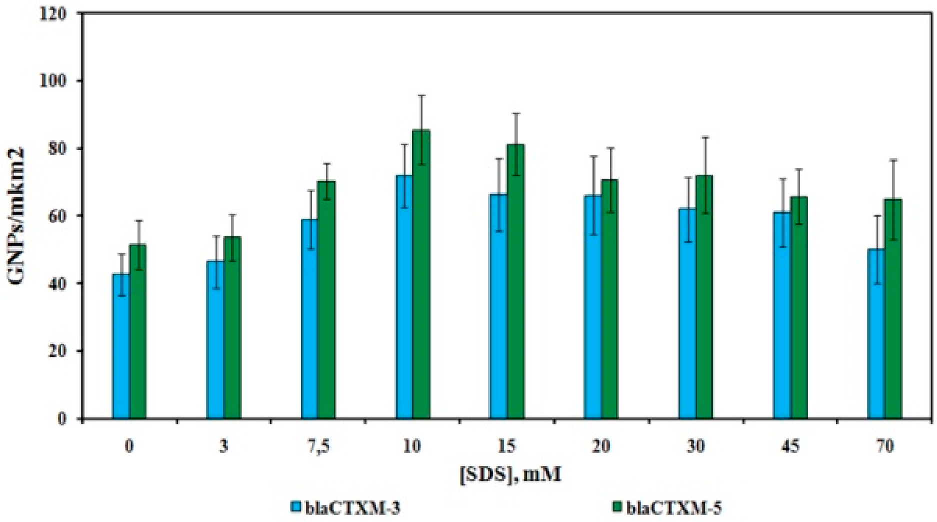

Figure S2. Effect of Mg^{2+} (**a**) and SDS (**b**) concentrations on the GNPs' density on specific microchip spots corresponding to immobilized Probe A and Probe CTX-M after the hybridization with100 pM target DNA, containing *bla*CTX-M-3 and *bla*CTX-M-5.

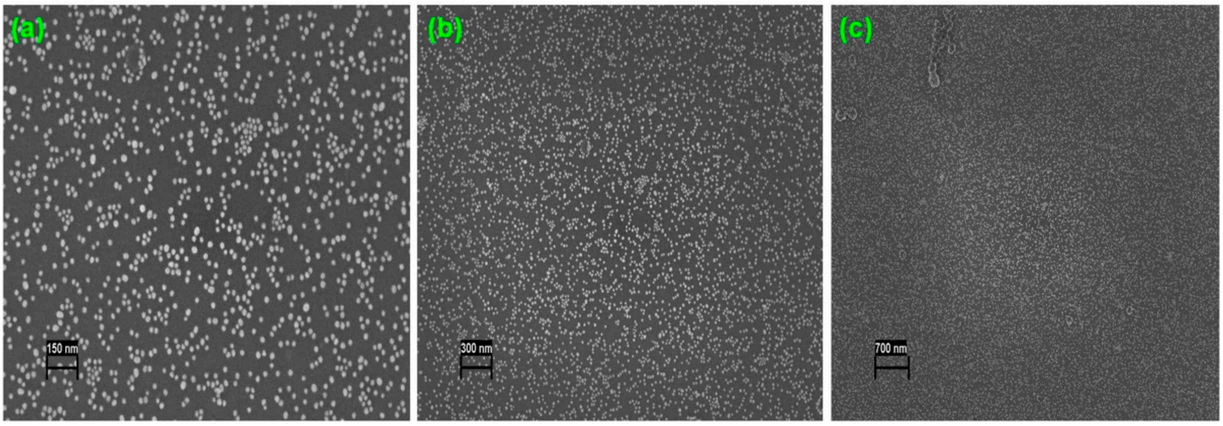

Figure S3. SEM images of the specific microchip spots after hybridization with 500 pM short target Probe C obtained at different magnifications of the microscope: 150KH (**a**), 75KH (**b**), 35KH (**c**).
